# Analysis of Sports Video Intelligent Classification Technology Based on Neural Network Algorithm and Transfer Learning

**DOI:** 10.1155/2022/7474581

**Published:** 2022-03-24

**Authors:** Han Guangyu

**Affiliations:** Physical Education Institute, Xinxiang Medical University, Xinxiang 453003, Henan, China

## Abstract

With the rapid development of information technology, digital content shows an explosive growth trend. Sports video classification is of great significance for digital content archiving in the server. Therefore, the accurate classification of sports video categories is realized by using deep neural network algorithm (DNN), convolutional neural network (CNN), and transfer learning. Block brightness comparison coding (BICC) and block color histogram are proposed, which reflect the brightness relationship between different regions in video and the color information in the region. The maximum mean difference (MMD) algorithm is adopted to achieve the purpose of transfer learning. On the basis of obtaining the features of sports video images, the sports video image classification method based on deep learning coding model is adopted to realize sports video classification. The results show that, for different types of sports videos, the overall classification effect of this method is obviously better than other current sports video classification methods, which greatly improves the classification effect of sports videos.

## 1. Introduction

As a branch of artificial intelligence, more and more different methods are emerging with the increasing development of theory, and they play an important role. Deep learning is one of the more popular areas. The development of DNN is driven by the continuous breakthroughs and innovations of deep learning. DNN is the most commonly used architecture in deep learning [1]. The concept of deep learning was first proposed by Hilton et al. in 2006. In recent years, it has shined in various fields, making it a hot topic in the AI field [2]. The main idea of deep learning is to simulate the neural structure of the human brain. It is hoped that the machine can process the received data like a human. The realization process is to extract features from the data through multiple nonlinear stages [3]. However, when we actually want to solve a problem by using machine learning algorithm, whether we use shallow machine learning algorithm or deep learning algorithm, we are faced with some practical conditions, such as the lack of data and information and the difference between training data and practical application data, resulting in poor model construction and poor practical application effect [4]. This is because traditional machine learning requires that the data of learning and the data of actual application scenarios should meet the same statistical characteristics, and it means that whenever there is a new application scenario, it is necessary to collect enough annotation information to make the traditional machine learning methods play an effective role, which will consume a lot of manpower, material resources, and time cost [5]. With the rapid development of machine learning in recent years, people pay more and more attention to this problem, and transfer learning, one of the learning methods to solve this problem, has become a very active research field [6].

The goal of transfer learning is to use existing knowledge to deal with problems in different but related fields, so as to realize the transfer of knowledge between related fields. It is an intelligent learning method similar to human basic learning ability. Just like in real life, we humans can judge whether the new problems we face are related to the previous accumulation and whether we can adopt the knowledge learned before through the knowledge we have learned or accumulated before [7]. Psychologists have found that, in order to realize transfer learning, it is certainly necessary for the two fields or learning materials to have common factors, and the transfer effect is proportional to the common factors, and the more the common factors, the better the transfer effect. Of course, there are common factors and different factors, which may also cause negative transfer and affect learning [8]. Sports video is a very important resource in video. Sports programs have hundreds of millions of loyal viewers all over the world. The classification of sports video has become the focus of many researchers. Sports video intelligent classification technology can not only intelligently classify and sort out massive sports video data and reduce people's workload, but also provide people with better spiritual enjoyment in daily life. It is also the basis of intelligent radio and television intelligent classification. Therefore, sports video intelligent classification technology can be widely used in sports video management, retrieval, query, and other fields and has broad development prospects and great value [9]. At present, the traditional transfer learning method is mainly to find a common feature space and map the data in two domains into the feature space by mapping function, so as to make the data distribution difference between the two domains as small as possible while maintaining the original characteristics of the data as much as possible and then train the classifier on these mapped data [10]. Existing sports video classification technologies pay more attention to the extraction of video features. It can be seen that feature extraction is the key to sports video classification. Researchers have proposed different feature models for classification in terms of motion, color, edge, etc., but different features. The role played in sports videos is different. Some features have a good classification effect. Obviously, the features extracted by these methods are not more expressive than those extracted by deep learning, and the deep transfer learning technology can better meet the end-to-end needs in practical applications. [11].

Video features mainly focus on motion, color, brightness, edge, texture, audio, etc. These features are mainly used in the classifier in a separate way, or in a simple linear fusion (horizontal); that is, different feature vectors are used in a linear combination according to the law. This fusion method improves the classification performance to a certain extent. However, most of them separate different features and ignore the semantic relationship between features [12]. In order to establish more connection and fusion between features, this paper proposes an intelligent sports video classification technology based on DNN and transfer learning and makes an in-depth study on the field of sports video classification. The third paragraph introduces the video region feature extraction and processing and migration learning video classification system architecture. Block brightness comparison coding and block color histogram are proposed, which reflect the brightness relationship between different regions in the video and the color information in the region. The fourth paragraph introduces the transfer learning of DNN. The algorithm of maximum mean difference (MMD) is proposed for migration learning. The fifth paragraph introduces the classification and experimental results of migration learning sports video images based on deep learning coding model. On the basis of obtaining the features of sports video images, the sports video image classification method based on deep learning coding model is adopted to realize sports video classification. In order to analyze the effectiveness of the method classification in the article, the sports videos are set to be figure skating, badminton, and yoga in order. Due to the supervised fine-tuning, the parameters of each layer of the deep learning network can be adjusted in the form of error backpropagation to optimize the classification effect.

Through the analysis of traditional transfer learning algorithm and DNN transfer learning algorithm, this paper studies a video classification transfer learning algorithm based on DNN. Innovation contributions include the following: (1) It solves the problem that the target domain data has no label and domain adaptation. (2) It provides different suggestions for automatic classification for the rapidly increasing amount of sports video data. (3) To improve the effect of sports video classification, a sports video classification method based on DNN and transfer learning is proposed. The classification accuracy, recall, and maximum value of this method are better than the comparison method, and a better classification effect of sports video is achieved.

## 2. Related Work

Many data mining and machine learning algorithms have achieved great success in many fields (such as classification, regression, and clustering). However, most of these algorithms have a common assumption: the training set and the test set are in the same feature space and obey the same distribution. Zhang et al. [13] point out that, with the passage of time and the adjustment of application scenarios, the training data collected before may be out of date. It is a pity to discard these out-of-date data, but the adjustment of scenarios leads to differences in statistical characteristics between training data and application data, resulting in poor model effect. Ma et al. [14] indicate that traditional machine learning does not have the ability to transfer learning. Whenever a new application scenario is encountered, the process of data collection, training, and verification will be carried out again. For example, every time we learn something from “nothing,” then every time we learn, we will spend a lot of energy. Migration learning can make full use of outdated data to ensure that the target model has better effect, thus reducing the cost of data collection in new target tasks. Moradzadeh et al. [15] proposed a method called joint distributed adaptation. They assume that the marginal distributions of the source and target domains are different from the conditional probability distributions, and the target domain has no data annotations. This method looks for a linear transformation, so that the edge distribution of the transformed data can be as close as possible, and the conditional probability distribution difference is also as small as possible. The method to measure the degree of the difference in the data distribution is still the maximum mean difference. Compared with TCA, this method requires iteration and the source domain needs to be labeled. However, the method proposed in [16] has a hyperparameter, that is, the number of subspaces, which means that the user cannot know how many intermediate points should be found. Mahmoud et al.[17] proposed a geodesic stream-core method to solve the problem of choosing several intermediate points.

Qiu et al. [18] show that principal components are used to reduce the dimensionality of video visual and audio features to describe video content, and time series of motion features are used to distinguish motion event classifications in football sports videos. Chen et al. [19] studied the classification of simple sports in sports videos by detecting some motion modes in video frames, such as running, jumping, translation, and zooming of the serve lens. Liu et al. [20] show that some knowledge in the source domain is obtained by training the source convolution neural network on the ImageNet dataset beforehand, and then the network is migrated and studied on the image classification datasets Calteech-101 and Catech-256, respectively, which improves the classification accuracy by 40% compared with traditional training methods. Zhang et al.[21] show that, by studying the transfer learning effect of different layer features of convolutional neural networks, it is found that the transfer learning ability of lower layer features is weaker than that of higher layer features. Wang et al. [22] studied the combination of video and audio features to classify videos and experimentally studied the effect of principal component analysis (PCA) on feature dimension reduction but failed to solve the correlation between features, and the fusion scheme of two types of features was slightly insufficient. Migration learning solves the problem of insufficient training of neural networks with small samples, while greatly reducing the training cost of the network. This paper takes sports video classification as the focus of research and applies convolutional neural networks to a broader field. Therefore, the research of transfer learning is one of the current development trends. Researchers are committed to finding better video features or using multifeature fusion methods to classify videos, while improving the performance of the classifier and the accuracy of classification.

## 3. Video Classification

### 3.1. Extraction and Processing of Video Area Features

**Figure 1:**
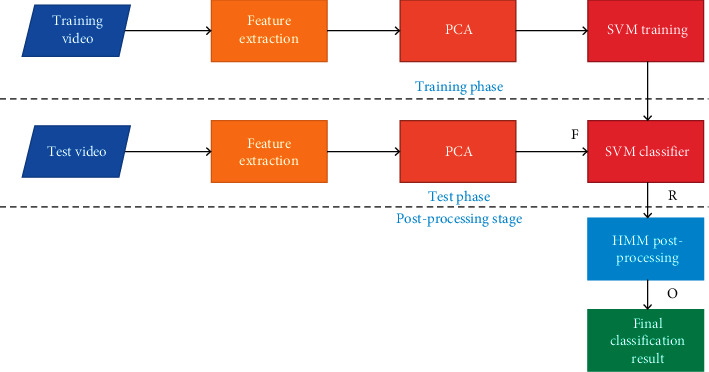
Video classification frame diagram.

Video area features are divided into (BICC) and block color histograms, which reflect the brightness relationship between different areas in the video and the color information within the area. The algorithm framework is shown in Figure 1.

For each type of video, the brightness distribution of the frame is relatively uniform and there is a certain brightness difference between the target in the frame and its surrounding environment. The human visual system distinguishes the object by the brightness difference between the target in the frame and its surrounding environment, and BICC is obtained by comparing the average brightness between blocks [23]. BICC reflects the brightness relationship between intraframe blocks and has strong anti-interference ability for different video segments of the same kind of video due to factors such as brightness and darkness.

Assuming that the frame size is *M*×*N*, each frame is divided into log×seven blocks, and the size of each block is *h*×*u*, where *h* = *M*/*k*, *u* = *N*/*k*, and *x*_*i*_ represents the i-th pixel in the block The brightness value of each block is $\bar{X\left(l\right)},l\in \left[1,k\times k\right]$; then

(1) $$\begin{array}{c} \bar{X\left(l\right)}=\sum_{i=1}^{h\times u}\frac{x_{i}}{\left(h\times u\right)}. \end{array}$$

If the block brightness comparison coded value is expressed as *y*, the result of the brightness comparison between the m-th block and the nth block in a frame can be expressed as (2), where 1 < *m* < *K*×*K*, 2 < *n* < *K*×*K*-1.

(2) $$\begin{array}{c} y\left[\left(m-1\right)\times \left(k\times k\right)+n+\frac{m\left(m+1\right)}{2}\right]=\left\{\begin{array}{cc} 1 & \text{if}\bar{X\left(m\right)}>\bar{X\left(n\right),}\\ 0 & \text{otherwise.} \end{array}\right. \end{array}$$

According to formula (2), the frame can be coded by 0 and 1 according to the block brightness mean value comparison.

For some videos, there is no obvious difference in brightness, but there is a significant difference in color distribution, such as volleyball and table tennis videos, which can be used for video classification by dividing video frames into blocks and counting their color histograms. The extraction process of block color histogram is as follows.

Assuming that the frame size is *M*×*N*, the frame is divided into *k*×*k* blocks, and the size of each block is *h*×*u*, where *h* = *M*/*k*, *u* = *N*/*k*, and *x*_ir_, *x*_ig_, and *x*_*ib*_, respectively, represent the i-th block in the block. The value of the *R*, *G*, and *B* components corresponds to the pixel point, *Р* represents the number of straight squares, and HIST_*m*,*n*,*p*_ represents the color histogram of the nth block in the frame, where *m* ∈ {*r*, *g*, *b*}, *n* ∈ [1, *k* × *k*], *p* ∈ [1, *P*], *p*_*m*_ represent the quantization range of the color component *m* corresponding to the *p* square, but

(3) $$\begin{array}{c} {\text{HIST}}_{m,n,p}=\sum_{i=1}^{h\times u}\left(x_{i,m,n}\in p_{m}\right). \end{array}$$

Features are extracted based on video frame blocks. When there are more block partitions and more histograms in the BlockHist histogram, the dimension of the extracted feature vector is higher. Therefore, PCA is used to reduce the dimension of the extracted features to speed up the processing.

### 3.2. Migration Learning Video Classification System Architecture

Convolutional neural network (CNN) is a neural network architecture for image classification, which usually includes convolution layer and pooling layer. The loss function and accuracy are reported on the test set of each CNN architecture. Loss function: how far is the difference between the prediction and the actual results? The greater the predicted value, the worse the accuracy of the model fitting data points. Accuracy of test set is the accuracy of the model's prediction of test set data.

**Figure 2:**
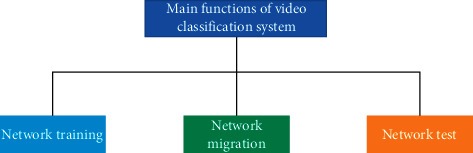
The overall framework of the system.

With the development of artificial intelligence technology, video classification technology is needed in various fields, such as face recognition, video location, and so on. On the one hand, with the advent of the Internet age, although the available target domain data that can fully meet the target tasks cannot be collected, enough original digital videos that are similar or related can be collected, and it is easier to train a source convolutional neural network that can better complete the video classification tasks [24]. In addition, DNN has gradually become a research hotspot in recent years, and the convolutional neural network, as a star in the field of video classification, has also made progress that cannot be ignored. Compared with other traditional video classification methods, it has a better classification accuracy. Therefore, most of the current fields are keen to complete video classification tasks through deep convolutional neural networks from many aspects such as processing speed, classification accuracy, and user experience. According to the functions of video classification related systems, the realized video classification system can be divided into three parts, namely, network training, network migration, and network testing [25]. The overall functional framework of the system is shown in Figure 2.

Network migration module is the core part of the whole system. For convolution models that are already available, the network training module can be almost unused, loaded directly with existing parameters, and then used. The migration module also has superparameter settings, which include not only the learning rate and iteration rate, but also the ratio of convolution layer and full-connection layer parameter changes and the K selection function. Task mapping is the mapping of the source and target tasks when changing the top-level classifier structure of the source domain model according to the target domain tasks, which makes the modification of the network more reasonable. The process of retraining the new network by the fine-tuning function is similar to that of the network training model, except that K selection algorithm is added in the process, and only some parameters are adjusted. The network test module has an evaluation method for the constructed convolutional neural network, so as to know whether the model meets the task research and how to modify the network.

**Figure 3:**
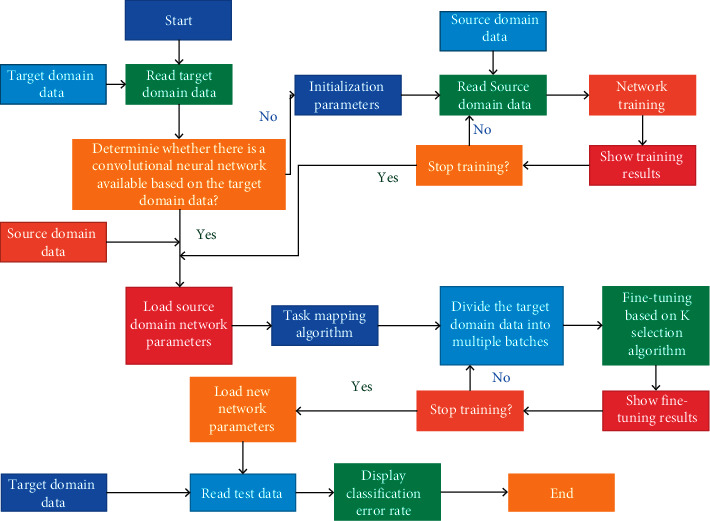
Work flowchart of video classification system.

The main workflow of migration learning video classification system is the training, migration, and testing process of convolutional neural network, in which whether the training process is carried out depends on whether there are trained available model parameters. The following describes the workflow of the system in the form of a flowchart, as shown in Figure 3.

## 4. DNN Transfer Learning

Fully connected neural network model (DNN) is also called deep neural network in some cases. Different from traditional perceptron, each node has an operation relationship with all nodes in the next layer, which is the meaning of “fully connected” in the name. The middle layer in the above figure also becomes a hidden layer. Fully connected neural network usually has multiple hidden layers. Adding a hidden layer can better separate data. However, too many hidden layers will also increase the training time and produce overfitting.

On the other hand, since the last feature of a neural network, such as the last layer of a convolution layer, must be related to a particular dataset, assuming that the last layer of the network is the softmax layer and that the entire network has been trained on a dataset, the output of the softmax layer must be closely related to that dataset, so make this feature special [26].

The first two modules of VGG13 network can be fixed (not counting pooling layer, but the first four layers) because the first few layers of DNN trained on image data set are all common features. These network layers have been trained on ImageNet data set and can be used to extract common features on source domain and target domain [27]. The domain adaptation problem is difficult to solve because the target domain data has no label. In order to solve this problem, many methods try to limit the error value of the target domain to the error of the source domain plus a difference value. MMD is one of the methods, and its formula is

(4) $$\begin{array}{c} \text{MMD}\left[F,p,q\right]=\left[\underset{f\in F}{\sup}\left(E_{p}\left[f\left(x\right)\right]|-|E_{p}|\left[f\left(y\right)\right]\right)\right]. \end{array}$$

Among them, F is the space where the function *f* is located, the distribution of *x* is *p*, and the distribution of *y* is *q*. Equation (4) means the upper bound of the difference between the expected value *E*_*p*_[*f*(*x*)] and *E*_*q*_[*f*(*y*)] after random projection of the data *x* on the source domain and the data *y* on the target domain through the function *f*. Obviously, when *p* = *q*, that is, when the distributions of *x* and *y* are the same, no matter how the function *f* is projected, the expectation will be the same, so in the case of *p* = *q*, MMD is 0. However, if *p*_q, and the function space F is rich enough, the MMD will never be zero. Its value is determined by the function in function space F which makes the distribution difference between the *X* and Y projections the greatest. It is important to note that if the function space F is too rich and *f* has too many possibilities to take values, MMD can easily be infinite, so the function space F needs to be constrained. F is proved to be the best when F is the unit ball in the reproducing kernel Hilbert space (i.e., space less than or equal to 1 from the origin). At this point, the formula for MMD^2^ becomes

(5) $$\begin{array}{c} {\text{MMD}}^{2}\left[F,p,q\right]=\left[\underset{{\left\|f\right\|}_{H}\leq 1}{\sup}\left(E_{x}\left[f\left(x\right)\right]\right)-E_{Y}\left[f\left(y\right)\right]\right],\\ \;={\left[\underset{{\left\|f\right\|}_{H}\leq 1}{\sup}{\langle \mu_{p}-\mu_{q},f\rangle }_{H}\right]}^{2},\\ \;={\left\|\mu_{p}-\mu_{q}\right\|}_{H}^{2}. \end{array}$$

An important property of (5) is that *p*≠q if and only if MMD^2^=0. Because a single kernel function may not be optimal, if a single kernel is used, it can be Gaussian kernel or linear kernel, but the disadvantage is obvious: it is impossible to know which kernel is good. Therefore, multikernel maximum mean error (MK-MMD) is used for feature mapping, and the kernel is weighted by *m* different Gaussian kernels, and the weight is *β*_*u*_, and the formula is

(6) $$\begin{array}{c} K=\left\{k=\sum_{u=1}^{m}\beta_{u}k_{u}:\beta_{u}\geq 0,\forall u\right\}. \end{array}$$

The restriction on the coefficient *β*_*u*_ ensures that the obtained *k* is unique, so that the measurement method for the difference between the source domain and the target domain is determined to be MK-MMD.

**Figure 4:**
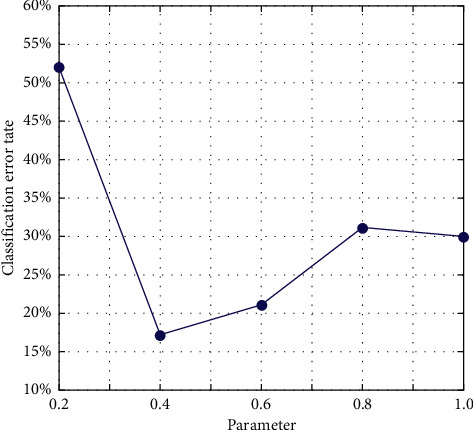
The impact of K selection on migration results.

**Figure 5:**
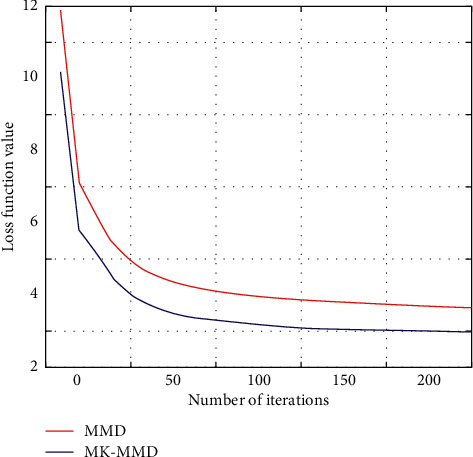
Two transfer learning loss functions.

In general convolution networks, the characteristics will eventually change from general to special before the last layer, and this transformation will become larger and larger as the number of layers of the network goes deeper to the full-connection layer. In other words, deep networks are almost specialized by specific tasks in the full-connection layer. If there is not enough data in the target domain for supervised learning, the full-connection layer cannot be trained directly in the target domain. Since the source domain is labeled, train the entire network on the source domain to improve its accuracy on the source domain. At the same time, use MK-MMD to make the data on the source domain be on the output of the fully connected layer and the target domain. The output difference of the data on the fully connected layer is as small as possible, so as to reduce the distribution difference between the data of the source domain and the data of the target domain after feature mapping, so as to achieve the purpose of migration learning. The experimental results are shown in Figures 4 and 5.

**Figure 6:**
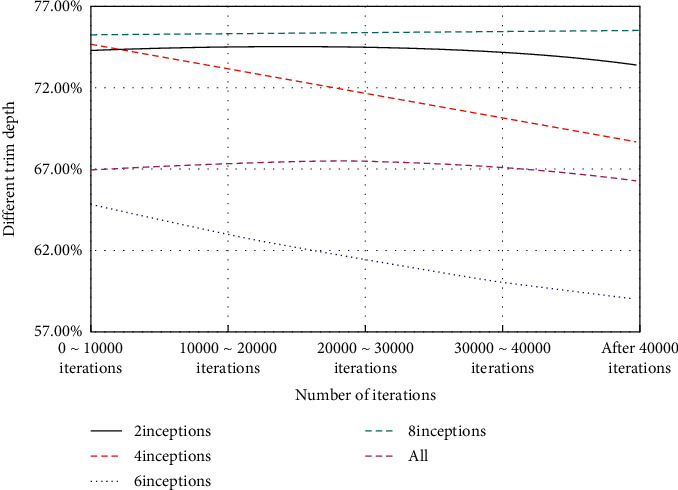
The accuracy of the FOOD101 GoogLeNet model with different fine-tuning depths in different training stages.

As shown in Figure 6, it can be seen that, in different stages of training, the accuracy of models with different fine-tuning depths is ranked consistent.

**Figure 7:**
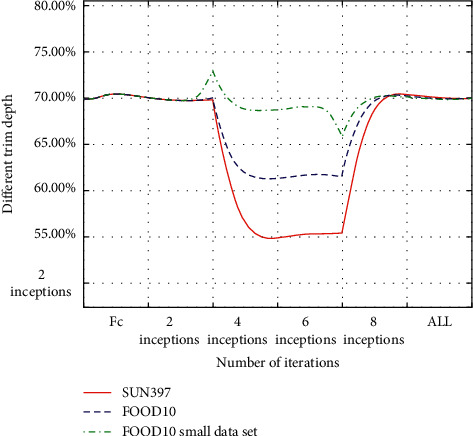
The accuracy of the three data sets under different fine-tuning depths of GoogLeNet.

**Figure 8:**
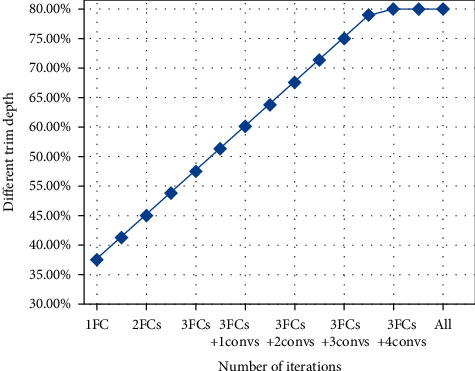
The accuracy of FOOD101 under different fine-tuning depths of Alex Net.

After carefully observing and analyzing the fine-tuning results at different depths, we find that the highest accuracy will first increase with the deepening of fine-tuning layers, then reach the highest at a certain depth, and then gradually decrease, showing a single peak curve. As shown in Figures 7 and 8, it can be observed that each curve has only one peak. For example, the peak of the GoogLeNet model of SUN397 is 4inceptions, and the peak of the GoogLeNet model of FOOD101 is 8inceptions. The peak of the AlexNet model of FoOD101 is 3 FCs+3convs. This shows that when starting the comparison from the smallest depth, once the highest accuracy rate of the next larger depth is not higher than the previous one, then the previous smaller depth is the best fine-tuning training depth. This kind of accuracy does not fluctuate with the change of the fine-tuning depth and is very suitable for iteratively finding the optimal solution without falling into the local optimum.

So you can think of an iterative approach to finding the best fine-tuned training depth. However, this method does not utilize the model weights that have been previously trained for each additional depth but instead retrains a model for a new fine-tuning depth.

**Figure 9:**
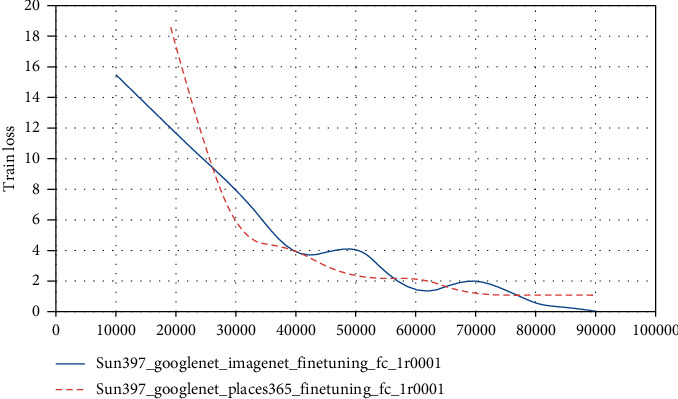
Train loss change curve during training.

**Figure 10:**
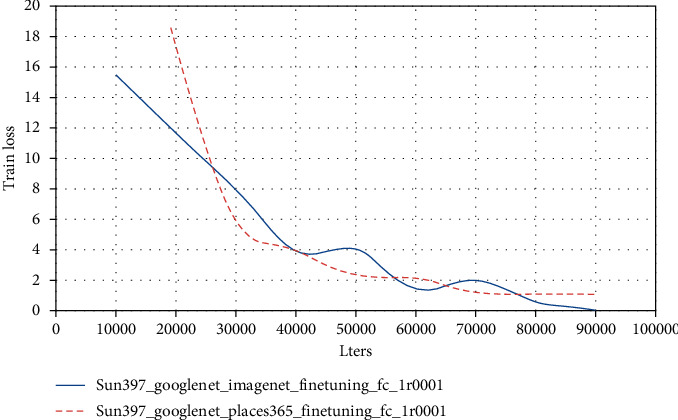
Test accuracy change curve during training.

According to the experimental results in Figures 9 and 10, using Places365_GoogLeNetfeature on SUN397 data set has better fitting effect on training set than ImageNet_GoogLeNetfeature and has higher accuracy on test set. Places365 is a scene dataset with 1.8 million pictures, SUN397 is also a scene dataset, and ImageNet is a large image dataset related to objects. Therefore, the more similar the new data is to the data used in the pretraining model, the higher the accuracy of the model after transfer learning.

## 5. Sports Video Image Classification and Experimental Results

### 5.1. Transfer Learning Sports Video Image Classification Based on Deep Learning Coding Model

On the basis of acquiring the characteristics of the sports video image's key chain frame, the sports video image classification method based on the deep learning coding model is adopted to realize the sports video classification. The specific detailed process is as follows:

1. Set the feature library of sports video images to be linked to *G*={*g*_1_, *g*_2_, ⋯, *g*_*m*_}, and *M* represents the number of features
2. Use unsupervised restricted Boltzmann machine (RBM) to encode *G*; use CD algorithm to obtain convergence parameter codebook and become a complete dictionary
3. Use the kind label information of training data to spread the errors of the dictionary acquired by learning nonforward, supervise the learning of RBM network, and then optimize the acquisition of G-code to get the optimized visual dictionary and description coefficient
4. Rub the support vector machine classifier with the deep learning coding vector of the training sports video image to realize sports video classification

G is coded by using multilayer restricted Boltzmann machine and becomes an exemplary visual dictionary. According to the spatial information of *G*, the neighboring *G* features are set as the input of RBM, and the RBM is trained by CD fast algorithm to obtain the hidden stack features. Then, the adjacent hidden layer features are regarded as the input of the lower RBM, and the output dictionary is obtained. Among them are *ϖ*_1_ and *ϖ*_2_. Belonging to the connection weight of RBM, RBM has a visible layer and a hidden layer, and neurons based on the same level in RBM do not have a connection relationship. During network training, the hidden layer and the explicit layer of the RBM are connected according to the conditional probability distribution. The conditional probability of the explicit layer and the hidden layer is

(7) $$\begin{array}{c} q\left(s_{i}|y\right)=\text{sigmoid}\left(c_{i}+\sum_{j=1}^{i}\varpi_{ji}y_{i}\right),\\ q\left(y_{i}|s\right)=\text{sigmoid}\left(b_{i}+\sum_{j=1}^{i}\varpi_{ji}s_{i}\right). \end{array}$$

In the formula, *y*_*i*_ and *s*_*i*_ describe the feature layer and encoding layer of sports video in turn, that is, the explicit layer and the hidden layer in RBM.

Using CD algorithm, RBM is quickly learned to improve the convergence efficiency of parameters, and the amount of update to obtain weights is *ϖ*_*ji*_:

(8) $$\begin{array}{c} \Delta \varpi_{\text{ji}}=\phi \left({\langle y_{i}s_{i}\rangle }_{\text{data}}-{\langle y_{i}s_{i}\rangle }_{\text{model}}\right). \end{array}$$

Among them, *ϕ* describes the learning speed. Using CD algorithm can get the latest parameters of sports video features until the parameters converge and get the initial visual dictionary.

### 5.2. Experimental Results

In order to analyze the effectiveness of the method classification in the article, the sports videos are set to be figure skating, badminton, and yoga in order.

When the method in the test article recognizes the three types of sports videos of figure skating, badminton, and yoga, the comprehensiveness of the key frames of the video images is extracted, and the missed recognition rate is the evaluation index. The results are shown in Table 1.

The data in Table 1 shows that when the method is used to identify the three types of sports videos: figure skating, badminton, and yoga, the average miss rate of extracting video image keyframes is less than 0.03, which indicates that the method in this paper can extract the key frame features from sports video images in an all-round way. Three sports videos were imported into the Caltech image collection to test the interference of different visual point sizes on the methods in this paper. With the increasing scale of sports video visual dictionary, the classification accuracy of the proposed method also increases. When the scale of sports video visual dictionary reaches 2000 MB, the classification accuracy of the proposed method is stable at 0.98. In order to test the influence of supervised fine-tuning on the classification effect of the proposed method, there are fine-tuning and no fine-tuning in the test. There is a significant difference between supervised fine-tuning and unsupervised fine-tuning. Supervised fine-tuning can improve the classification accuracy of methods in the text. This is because supervised fine-tuning can use the form of error backpropagation to adjust the parameters of each layer of the deep learning network to optimize the classification effect.

## 6. Conclusions

By analyzing the traditional transfer learning algorithm and DNN transfer learning algorithm, this paper studies a DNN based video classification transfer learning algorithm and designs it as a video classification system based on convolutional neural network. The first two modules of vgg13 network can be fixed because the first several layers of DNN trained on the image data set are common features. These network layers have been trained on ImageNet datasets and can be used to extract common features on source and target domains. Because the target domain data has no label, the domain adaptation problem is difficult to solve. How to automatically classify these sports videos has become a hotspot and difficulty in current research. In order to improve the effect of sports video classification, a sports video classification method based on DNN and transfer learning is proposed. In the classification process of this method, supervised fine-tuning is introduced to improve the classification accuracy of this method. The experimental results show that, for figure skating, badminton, and yoga sports videos, the classification accuracy, recall, and maximum value of this method are better than the comparison method, and a better sports video classification effect is achieved.

## Figures and Tables

**Table 1:** The key frame miss rate of sports video images of the method in the article.

Number of extractions	Figure skating	Badminton	Yoga
1	0.03	0.03	0.02
2	0.03	0.03	0.02
3	0.02	0.03	0.02
4	0.02	0.03	0.03
5	0.02	0.04	0.03
6	0.03	0.04	0.03
7	0.02	0.03	0.04
Mean value	0.02	0.03	0.03

## Data Availability

The experimental data used to support the findings of this study are available from the corresponding author upon request.
